# Rabies Virus Transmission in Solid Organ Transplantation, China, 2015–2016

**DOI:** 10.3201/eid2309.161704

**Published:** 2017-09

**Authors:** Shuilian Chen, Heng Zhang, Meiling Luo, Jingfang Chen, Dong Yao, Faming Chen, Ruchun Liu, Tianmu Chen

**Affiliations:** Changsha Center for Disease Control and Prevention, Changsha, Hunan, China

**Keywords:** Rabies, solid organ transplantation, transmission, China, viruses, meningoencephalitis, meningitis/encephalitis

## Abstract

We report rabies virus transmission among solid organ transplantation recipients in Changsha, China, in 2016. Two recipients were confirmed to have rabies and died. Our findings suggest that more attention should be paid to the possibility of rabies virus transmission through organ transplantation for clinical and public health reasons.

In 2016, Zhou et al. reported a case of rabies virus transmission in China that was probably a result of organ transplantation (*1*). We report on rabies transmission that occurred among solid organ transplant recipients in Changsha, China, during December 2015–January 2016.

In November 2015, the donor, a previously healthy boy, showed development of fever, insomnia, and agitation. On day 6 of infection, these symptoms progressed, and he was sent to a healthcare center. At this time, he experienced weariness, no desire to drink water, poor appetite, and panic. One day later, he began vomiting, and was admitted to a local hospital (hospital A), where he exhibited anemophobia, convulsions, limb rigidity, and hypersalivation. The patient was moved to hospital B (days 7–14) in Changsha. At admission, some examination findings indicated a possibility of viral encephalitis (Technical Appendix Table 1). Subhypothermia hibernation therapy and assisted ventilation were administered within 72 hours of admission, and the patient’s vital signs became stable. On day 10, hyponatremia was observed, and on day 11, the patient again became febrile and tachycardic, with hypertensive abdominal distention and alimentary tract hemorrhage. On day 13, viral encephalitis was diagnosed, and rabies was suspected. However, rabies virus antibody tests performed on serum samples by using ELISA yielded negative results. 

On day 14, the patient was transferred to hospital C, where he became comatose and was declared brain dead. Permission was granted for organ donation, because no specific pathogen had been detected and China’s organ transplant policy allows for organ donations in cases of viral encephalitides without laboratory-confirmed pathogens. Rabies virus–specific binding antibodies were also not detected in the serum samples. The donor had possibly been exposed to rabies but had not been vaccinated against rabies (online Technical Appendix). The patient’s kidneys and liver were removed for transplantation in hospital D in Changsha on the following day.

Kidney and liver transplantations were performed on December 10, 2015. Two female recipients in hospital D received the kidneys, and an 8-month-old girl in hospital E in Shanghai, China, received the liver. No other organs were collected or transplanted from this donor. The surgeries were all uneventful, and the recipients were discharged after transplantation. However, all 3 recipients were eventually readmitted to the hospital with complex symptoms (Figure). 

**Figure:**
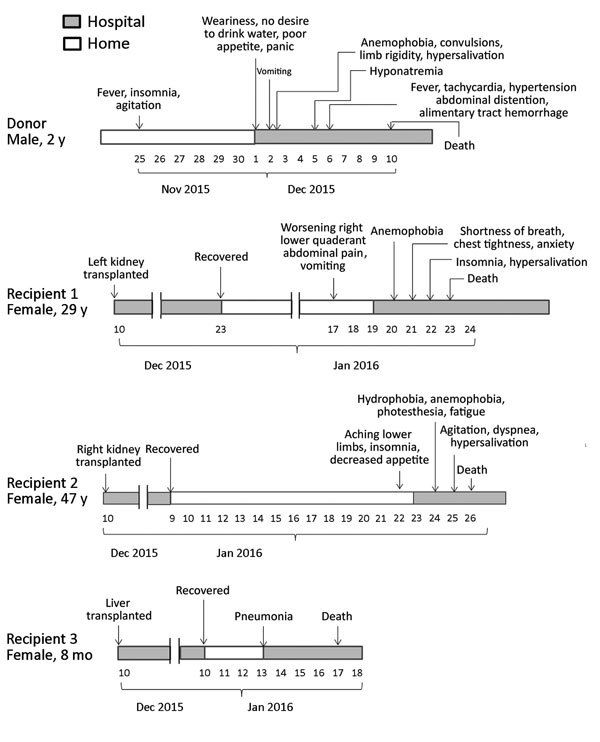
Clinical course of a donor and 3 recipients in a rabies outbreak associated with solid organ transplantation, Changsha, China, 2015–2016.

On the 40th day after transplantation, the left kidney recipient (29 years of age) revisited hospital D, reporting worsening right lower quadrant abdominal pain and vomiting. In the next 3 days, she successively had anemophobia, shortness of breath, chest tightness, anxiety, insomnia, and hypersalivation. During hospitalization, her blood pressure was as high as 248/148 mm Hg. On her 7th day in the hospital, she became comatose and then died. 

On the 43rd day after transplantation, the right kidney recipient (age 47 years) developed aches in her lower limbs, insomnia, and a decreased appetite. She was readmitted to hospital D the next day. On the 3rd day after admission, she exhibited hydrophobia, anemophobia, photesthesia, and fatigue. She showed agitation, dyspnea, and hypersalivation on the 4th day; she became comatose and died 1 day later. 

The liver recipient was readmitted with pneumonia on the 34th day after transplantation and died of asphyxia and multiple organ failure within 5 days. This patient did not show any signs or symptoms of rabies or encephalitis. 

None of the recipients had been exposed to potentially rabid animals or had been vaccinated previously for rabies (online Technical Appendix). Both kidney recipients tested positive for rabies virus (Technical Appendix Table 2). The genome sequences of the rabies virus isolates from the right kidney recipient (isolate no. CCS1501H) were ≈11 kb nucleotides in length and belonged to the China I lineage. No testing for rabies was done on the donor or on the liver recipient.

In the past 10 years, rabies transmission by solid organ transplantation has been described occasionally worldwide (*2*–*4*). Hence, rabies transmission through organ transplantation is a clinical and public health concern. To prevent future cases such as this, we recommend that patients with unexplained encephalitis or mental status changes should not be used as organ donors even if tests for some infectious causes of encephalitis are negative. In addition, if rabies is suspected in the donor after organs have been transplanted, the recipients should also not be used as organ donors. An antibody test is not the ideal choice for the diagnosis of rabies virus and by itself cannot reliably exclude rabies from the differential diagnosis. For this reason, a combination of multiple techniques, preferably direct fluorescent antibody test and reverse transcription PCR, should be used before organ transplantation, especially when the donor is suspected of having rabies or a potential exposure to rabies. In addition, if a patient has meningoencephalitis of unknown cause, a specific epidemiologic and laboratory evaluation should be performed to conclusively rule out rabies as a cause of illness before organ donation.

Technical Appendix Epidemiologic information about donor and the 2 kidney transplant recipients in a rabies outbreak associated with solid organ transplantation, Changsha, China, 2015–2016.
