# Supplementary material for: Rabies Virus Transmission in Solid Organ Transplantation, China, 2015–2016
Source: Emerg Infect Dis. 2017 Sep;23(9):1600–2. doi: 10.3201/eid2309.161704 (PMC5572883; doi:10.3201/eid2309.161704)
Supplement: Technical Appendix — Epidemiologic information about donor and the 2 kidney transplant recipients in a rabies outbreak associated with solid organ transplantation, Changsha, China, 2015–2016. [file 16-1704-Techapp-s1.pdf]

Article DOI: https://doi.org/10.3201/eid2309.161704

Rabies Virus Transmission in Solid Organ Transplantation, China, 2015–2016

Technical Appendix

Epidemiologic Information on Donor and the 2 Kidney Transplant Recipients in a Rabies Outbreak Associated with Solid Organ Transplantation, Changsha, China, 2015–2016

Family members reported that the donor had been hurt outside the house 2 months previously, with 1 wound each on the forehead and occiput. The reasons for the injuries were unclear, and he had not been vaccinated for rabies. Our field investigation showed that many stray dogs lived in the donor's village; thus, the donor may have been bitten by a stray dog(s). Interviews with the kidney transplant recipients and their family members indicated that the recipients had neither been exposed to potentially rabid animals nor been vaccinated previously for rabies. Therefore, transplantation was a possible route of transmission.

Technical Appendix Table 1. Medical examination of the donor in hospital B

Date	Day of infection	Medical examination	
		Test	Outcome
Dec 2, 2015	7	Heart examination	Tachycardia (189 beats/min; normal range 100–120) with a gallop rhythm
		Cerebrospinal fluid analysis	Normal leukocyte count with normal differential<br>Decreased protein levels (136 mg/L; normal range 150–450)<br>Elevated chloride levels (136 mg/L; normal range 110–122)
		Routine blood count	Increased platelet count (382 × 10^9/L; normal range 125–350 × 10^9/L) and elevated leukocyte count (21.62 × 10^9/L, normal range: 3.5 – 9.5 × 10^9/L) with neutrophils representing 65.8% (normal range 40.0%–75.0%) and lymphocytes 28% (normal range 20.0%–40.0%)
Dec 5, 2015	10	Plasma electrolyte	Hyponatremia (sodium 116 mmol/L; normal range 135–145 mmol/L)

Date	Day of infection	Medical examination	
		Test	Outcome
Dec 6, 2015	11	Physical examination	Tachycardic (pulse 192; normal range 100–120) and hypertensive (blood pressure 170/110 mmHg; normal range 83/55 mmHg)
Dec 7, 2015	12	Computed tomography scan	No intracranial abnormalities

Technical Appendix Table 2. Laboratory tests for the rabies virus in a rabies outbreak associated with solid organ transplantation, Changsha, China, 2015–2016 *

Patient	Number of saliva samples specimens	Method 1	Outcome 1	Method 2	Outcome 2
Donor	NA	NA	NA	NA	NA
Recipient 1	1	RT-PCR	Positive for rabies virus	NA	NA
Recipient 2	3	RT-PCR	Positive for rabies virus	Whole-genome sequencing	Isolates belong to China I lineages
Recipient 3	NA	NA	NA	NA	NA

*NA, not available; RT-PCR, reverse transcription-polymerase chain reaction.

.
